# Long-term support referrals to enhance food security and well-being in older adults: Texas physicians and nurses on what works

**DOI:** 10.1007/s10389-022-01800-5

**Published:** 2023-01-23

**Authors:** Nandita Chaudhuri, Laila Hussain Alvi, Ashleigh Williams

**Affiliations:** Public Policy Research Institute, Texas A&M University, 4476 TAMU, College Station, TX 77843 USA

**Keywords:** Congregate meal programs, Food security, Social isolation, Healthcare providers, Long-term support services, Senior nutrition

## Abstract

**Aim:**

Senior participation in the congregate meal programs (CMPs) has alarmingly declined over the past decade in Texas as elsewhere in the nation. The purpose of this survey study was to identify the possible reasons for this decline from the viewpoint of the Texas physicians and nurses who are key in coordinating care and ensuring food security for the vulnerable older Texans by referring them to community-based long-term support services (LTSS).

**Subject and methods:**

The methodology adopted was an online panel survey of physicians and nurses from rural and urban Texas counties. Structured multiple-choice and open-ended questions primarily focused on provider referral processes, reasons for connecting older clients to CMPs, perceptions about various aspects of these programs, possible reasons for the decline in participation, suggestions to make the programs an integral part of the community-based LTSS referral system, and how to address the COVID-19 pandemic constraints on the programs.

**Results:**

As a majority of the healthcare providers surveyed were unaware of the CMPs in their communities, the study spotlighted an urgent need for a better-coordinated referral process centered on strategic marketing and awareness-building about the CMPs, including an extensive healthcare provider education component as well as an overall improvement in meal quality and variety.

**Conclusion:**

The study highlights a need for additional research so decision-makers better understand how to best disseminate information to healthcare providers to improve the referral mechanisms, increase the referrals, and enhance the overall CMP program quality to benefit the vulnerable food-insecure older adults.

## Introduction

The older American population in the United States is likely to increase by 112.2% from 2014 to 2060 (Colby and Ortman 2015). As the population of elderly individuals grows, so does the food insecurity amongst them. In 2019, 9% of older Americans were food-insecure and suffered from chronic health conditions such as asthma, heart failure, hypertension, and diabetes, leading to increased government spending on healthcare (Coleman-Jensen et al. 2014; Lloyd and Wellman 2015). In the past, many studies have shown that nutrition interventions are a cost-effective way to promote healthy living (Mower 2008; Lloyd and Wellman 2015; Greenlaw 2018). However, these interventions must incorporate the changing demographics, tastes, and preferences of older Americans to be successful (Colby and Ortman 2015).

The Older Americans Act Title III-C Congregate Meal Programs (CMPs) are an attempt to address food insecurity, hunger, malnutrition, and social isolation of older American adults, the fastest-growing population. Across the nation, however, participation in these programs has been declining (Mabli et al. 2017). While the population of adults aged 60 years and older increased by 2.4 million from 2005 to 2020, the Administration for Community Living’s (ACL) Aging Integrated Database (AGID) indicates that Texas served 66% fewer congregate meals in 2020 than it did in 2005 (Administration for Community Living [ACL] n.d.). This presents a unique problem for policymakers and aging stakeholders in modernizing the programs and facilitating greater participation.

Efforts have been made to identify program barriers and facilitators through ACL-funded Innovations in Nutrition Programs and Services (INNU) projects. One such project in Texas has been the 3-year (2019-2022) Texas Congregate Meal Initiative (TCMI). As part of the comprehensive gap analysis in this project, a key survey was conducted to understand the perceptions of the Texas physicians and nurses. These healthcare providers play a vital role in providing care coordination for older adults and connecting them with community-based long-term support services (LTSS). Prior to TCMI, no such statewide survey of healthcare providers existed either in Texas or in the nation. The purpose of this survey was to gain an overall understanding of the healthcare provider referral processes to CMPs in Texas and to grasp the provider perceptions about possible barriers and facilitators for the programs. It is hoped that findings from the survey will facilitate the integration of CMPs as successful components in LTSS for the Texas elderly and promote their well-being.

### Methodology

Health provider surveys are popular as special population surveys due to their cost-effectiveness and ability to cover large populations across geographic regions (Carter et al. 2020). As the first point of contact for individuals seeking medical assistance, health providers add valuable insight on access, quality, and delivery of healthcare. They also provide acumen on standards of health facilities, client attitudes towards health services, and discrepancies in healthcare services for the senior citizens in their communities (Centers for Disease Control and Prevention [CDC] n.d.). This information is crucial to public health professionals, policymakers, and researchers in implementing policies and strategies that improve community-based programs catering to seniors. The reliability and generalizability of health survey findings, however, are significantly affected by the methods used in conducting health surveys (McColl et al. 2001; Edwards 2010; Carter et al. 2020). Thus, choosing the right methodology in conducting health provider surveys is important. This section briefly describes the methodology used for the community health provider survey.

Because a key goal of CMPs is to address food insecurity in underserved low-income older adults who struggle with adequate access to nutritious meals, the survey targeted Texas physicians and nurses that serve Medicaid and Medicare patients, along with other patients, in rural and urban Texas counties. It was administered through Qualtrics, an online survey sample company, which served as the aggregator of an online panel and used four different panel partners for participant recruitment. An online panel is a group of people who have agreed to take surveys online in return for some monetary incentive. The company has over 100 million participants on its various panels with registered hard-to-reach users, and they participate in specific surveys in exchange for monetary incentives (Armstrong et al. 2021). The online panel survey was conducted from August 3, 2020, to August 16, 2020, and a total of 412 responses were collected.

### Survey objective

The key survey objectives were to understand how Texas physicians and nurses refer older American clients with nutrition and socialization needs to CMPs in the various communities they serve and to gather information on provider knowledge pertaining to CMPs as well as their perceptions of the meals provided at congregate meal sites. Nutritional interventions are crucial for older adults to lead a healthy lifestyle and avoid chronic health conditions in today’s rapidly expanding older American population (Lloyd and Wellman 2015), as food insecurity and malnutrition have significantly increased in this population over the past decade (Evans 2005). A study from 2020 showed that 6.8% of older adults were food-insecure, 2.6% of older adults had very low food security (Ziliak and Gundersen 2022), and 50% of older adults were malnourished (Greuling 2016). Older adults who are food-insecure tend to consume more low-calorie food, resulting in lower intake of required essential nutrients and leading to poor nutritional status and higher chronic health conditions (Lloyd and Wellman 2015).

The CMPs in Texas provide nutritionally balanced meals, nutrition education, and nutrition risk screening for Texans aged 60 years and older and their spouses, while encouraging socialization and better health through disease prevention and health promotion programs. In addition to meals, nutrition counseling and other activities to enhance social engagement and promote the well-being of the older adults within congregate settings might be available in some areas. In this context, it was important to know the Texas physicians’ and nurses’ perceptions about the facilitators and barriers for CMP participation by older adults in their communities. The survey was specifically designed to understand the LTSS referral process used by the physicians and nurses, their perceptions about various aspects of CMPs, and their opinions on what strategies might work in increasing older American client participation in CMPs.

### Survey instrument

The community health provider survey instrument was used to assess the perceptions of healthcare providers who serve older Americans within the state of Texas. The survey was divided into two subsections: initial screening and main content area. Initial screening questions facilitated appropriate sampling by identifying the respondents as practicing physicians in Texas, registered nurses, practicing licensed vocational nurses, or practicing advanced practice registered nurses in Texas. Respondents who did not belong to one of these groups were eliminated from participation in the survey, and those who belonged to one of the groups were asked whether they accepted Medicare or Medicaid. Respondents were also able to identify which county they served out of the 254 Texas counties.

The instrument included a total of 15 multiple-choice and open-ended questions. The multiple-choice questions focused on reasons for referring clients to CMPs, the overall referral processes in their service settings, channels through which older adults hear about CMPs, key perceptions about various aspects of CMPs, and reasons for the decline in CMP participation. The open-ended questions asked for suggestions of ways to make CMPs an integral part of their community-based support referral process and potential solutions to address the limiting impact of COVID-19 on senior participation in CMPs.

Survey language was carefully crafted to avoid any suggestive or ambiguous phrasing and emotional loading. Wherever possible, participants were given the choice of selecting multiple responses to gather all possible perspectives through questions that were clear, answerable, and unbiased. Staying away from complicated words and technical terms that might not resonate with the providers allowed the survey instrument to focus on what it intended to measure and ensured that the results obtained were truly reflective of the population under study (Boateng et al. 2018). The instrument integrated careful alignment of question stems and answer choices and avoided double-barreled questions which could make it impossible for respondents to answer accurately (Boateng et al. 2018). These steps were intended not only to enhance respondent experiences but also to ensure quality, usefulness, and reliability of responses. Questions were guided by comprehensive literature searches and internally tested several times before piloting and finally launching.

### Survey implementation

A panel survey methodology through Qualtrics was used for data collection because healthcare providers are a special population who are hard to reach through random sampling. Over the years, the online panel survey has become a common method of data collection for special populations, because the internet-linked devices (e.g., laptops, tablets, and smartphones) used by these populations allow larger geographies to be covered quickly and cost-effectively by ensuring anonymity compared to traditional methods of data collection (Craig et al. 2013; Hays et al. 2015). Currently, panel surveys are used in various fields including psychological research (Goritz 2007), social research (Tortora 2008), market research (Postoaca 2006; Comley 2007; Goritz 2010), medical research (Couper 2007), and election studies (Clarke et al. 2008).

For the present study, Qualtrics screened for physicians, registered vocational nurses, and advanced practice nurses in Texas from both urban and rural counties that served Medicaid and Medicare patients in Texas. Those who met the criteria and agreed to provide feedback received a survey link administered by Qualtrics. In addition to the panel respondents from Qualtrics, a survey link was shared with nutrition providers in Texas with the help of the Texas Health and Human Service Commission to generate additional responses beyond the Qualtrics panel. Data from both sources were later merged.

### Survey data

Quality control mechanisms were strictly implemented by Qualtrics to identify acceptable responses. Data collected were cleaned to ensure completeness and delete any duplication in responses. A total of 412 respondents completed the survey, of whom 204 were practicing physicians, 150 were registered nurses, 30 were licensed vocational nurses, and 28 were advanced practice registered nurses. The majority of the physicians and nurses served in counties classified as urban (347) while only 65 served in counties classified as rural, with Dallas and Harris counties being the predominant counties. Key insights from the study are presented in the following section.

## Results

A total of 412 respondents completed the questionnaire, of whom 49.5% were practicing physicians. The sample also included registered nurses (36.4%), licensed vocational nurses (7.3%), and advanced practice registered nurses (6.8%). Of those sampled, 84% of physicians and nurses served in Texas counties classified as urban, and 15.7% served counties classified as rural. Survey items addressed five broad categories pertaining to providers’ knowledge, behaviors, and perceptions related to CMPs and CMP referrals: (a) referral choices made by providers and intent of referrals, (b) the referral processes, (c) information sources about CMPs, (d) perceptions on CMP quality and participation decline, and (e) suggestions for increasing CMP referrals and usage. Results for each category are reported in the following sections.

### Referral choices and reasons for referring and not referring to CMPs

**Fig. 1:**
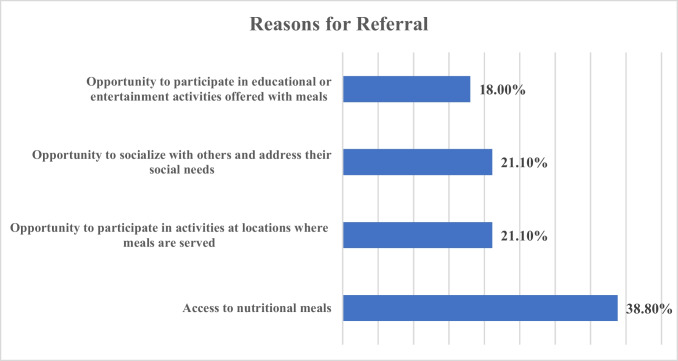
Reasons for provider referrals to congregate meal programs. Note: Respondents were permitted to select more than one option for this item; therefore, percentages will not add up to 100%

Survey results indicated that 39.8% (164) of the respondents have directly or indirectly (via a provider’s office) referred their patients to local CMPs in the past. Of those respondents, nurses were more likely to refer patients to CMPs (41.1%) than physicians (32.4%). When asked about the reasons for these referrals (see Fig. 1), providers selected the following reasons: to enable patients to have access to nutritional meals (38.8%), the opportunity to socialize with others and address their social integration needs (21.1%), and the opportunity to participate in activities at locations where meals are served (21.1%). The option least cited as a reason for referral was the opportunity to participate in educational or entertainment activities offered with meals (18.0%). There were no differences in responses found between rural- and urban-serving respondents who referred patients to the CMPs for any given reason. For physicians and nurses, the rank order of options most often selected as reasons for referrals was similar.

**Fig. 2:**
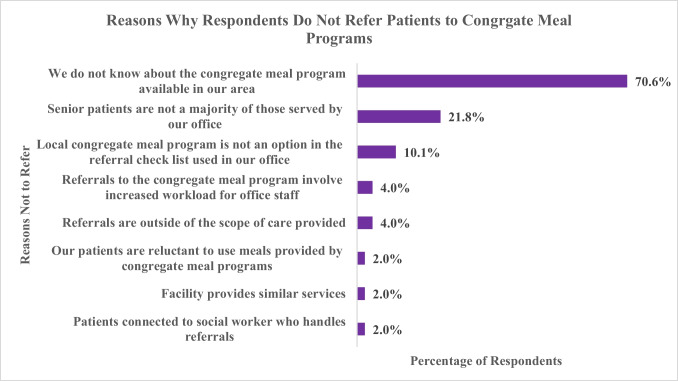
Reasons why providers do not refer patients to congregate meal programs. Note: Respondents were permitted to select more than one option for this item; therefore, percentages will not add up to 100%

Among the 60.2% (248) of respondents who had not referred patients to local CMPs, lack of awareness of the program was the reason most frequently cited by providers for not providing referrals (70.6%; see Fig. 2). Additional reasons included the following: senior patients not being a majority of the provider’s client base (21.8%), absence of local CMP as an option in the provider’s referral checklist (10.1%), increased workload for office staff (4.0%), similar community-based services already provided by the office (2.0%), and social workers handling referral coordination instead of the provider offices (2.0%). Although the reasons for not referring patients to CMPs did not differ much between the nurses and physicians, physicians were less aware of the existence of CMPs in the areas they served than the nurses.

### Referral processes

**Fig. 3:**
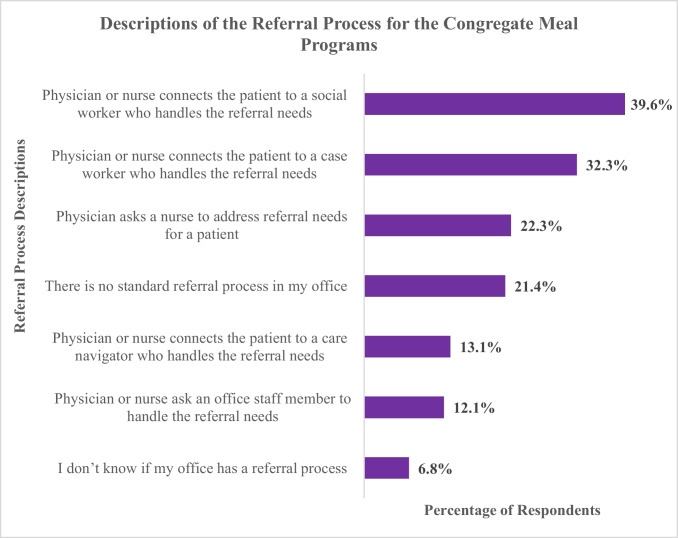
Descriptions of the referral process for the congregate meal programs. Note: Respondents were permitted to select more than one option for this item; therefore, percentages will not add up to 100%

A key reason for conducting the survey was to understand the existing referral processes used by physicians and nurses to refer patients to CMPs. Respondents were asked to select response choices that best described the referral processes used in their office. Of the respondents, 71.8% had a formalized referral process in their office, 21.4% did not have a standardized referral process, and 6.8% did not know whether their office had a referral process. Survey results revealed that the referral process varied widely across respondents who stated having a formalized referral process. As is shown in Fig. 3, 39.6% of the respondents indicated that a physician or nurse connected the patient to a social worker who handled the patient’s referral. Similarly, for 32.3% of respondents, a physician or nurse connected patients to a case worker who handled the referral needs. For 22.3% of respondents, a physician asked a nurse to address referral needs, and for 13.1% of respondents, a physician or nurse connected the patient to a care navigator in the office who handled their referral needs.

### Congregate meal programs information sources

Healthcare providers were also asked to indicate where they *think* the older adults in their communities hear about CMPs. The majority of respondents (70.9%) suggested that older adults hear about CMPs through family and friends. Respondents also thought that older adults hear about CMPs through community centers or older adult centers (66.5%), word of mouth (65.8%), and places of worship (58.7%). Few providers also indicated social media (16%) and online sources (11.7%) as avenues through which older adults in their community learn about congregate meal programs.

### Congregate meal programs quality and participation decline

Respondents were asked to rate the quality of the CMPs offered in their community. Twenty-six percent of participants rated the programs as good, 17.5% rated them as very good, and 53% rated them as excellent. An additional 15.3% indicated that the quality of the programs varies in the community: some are good, and some are not. Finally, 3.2% of participants rated the program as poor, while 21.6% of participants preferred not to rate the programs.

Respondents were also asked about the potential reasons for the decline in CMP participation by older Texans. Most respondents (81.8%) indicated lack of transportation to travel to the CMP locations as a potential reason for the decline. Fifty-eight percent stated that seniors might be reluctant to accept charity, and 49.4% thought that the stigma attached to free meals in group settings might be the reason older Americans choose not to participate in such programs. Other reasons included scheduling/timing challenges (31.1%), dietary restrictions (26.0%), concerns with the ambience of the settings where meals are served (24.3%), and poor quality of the meals served (19.9%).

Both urban and rural respondents stated that transportation is likely the largest barrier to CMP participation. However, the idea that some older adults do not feel frail or old enough to attend congregate meal programs ranked higher in terms of frequency of responses for respondents serving urban counties than for respondents serving rural counties. Although there were no significant differences between physicians and nurses in identifying a reason for why older adults may not want to participate in the CMPs, a larger percentage of nurses (66.3%) than physicians (49.5%) thought the reluctance to accept charity may be a reason why some seniors do not participate in the CMPs.

### Suggestions to improve CMP referrals and participation

**Fig. 4:**
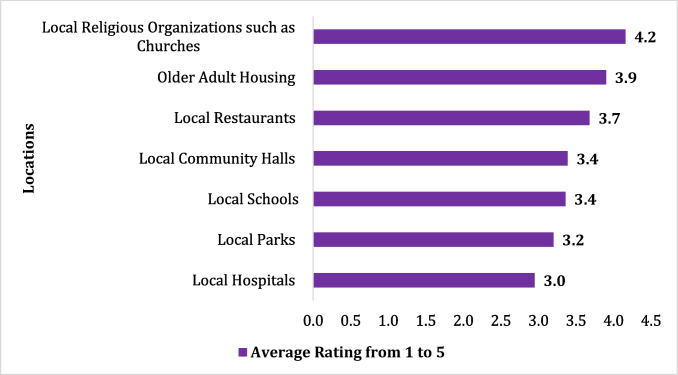
Ease of access for congregate meal programs by location

The congregate meals are generally offered in senior centers, elderly housing facilities, schools, churches, restaurants, and other community settings in Texas as in other parts of the nation. Participants were asked to rank the ease of access for various possible CMP community settings with the help of a 1-to-5 rating, where 1 meant most difficult to access and 5 meant easiest to access. As is presented in Fig. 4, religious organizations such as churches were ranked as the most easily accessible locations, followed by older adult housing and local community halls. Local hospitals and local parks were ranked as the most difficult to access of the settings listed. The rank order of ratings for ease of access did not vary between the Texas counties classified as rural or urban.

Participants were also asked about their perception of CMP benefits and suggestions for increasing CMP referrals and usage. A majority of participants said that CMPs helped address the economic needs of older adults by providing meals (4.3 rating out of 5). Respondents also stated that these programs helped fulfill the nutritional, dietary, and socialization or social connection needs of the elderly (4.2). The lowest rating for CMPs was in addressing the entertainment needs (3.5) of the elderly.

Respondents were further asked to provide three key suggestions about what could be done to make the congregate meal program an integral part of seniors’ community-based support. Almost half (41.6%) of all the responses indicated that better marketing and community outreach would help to make CMP an integral part of the referral process used by health providers for the senior population. These responses were in line with the studies done in the past that also suggested marketing and community outreach as effective ways to attract older adults to CMPs (Hoerr et al. 2016; Jiang et al. 2017; Schultz et al. 2021). Improved marketing would also assist in combating the negative perceptions of congregate meal sites (Hoerr et al. 2016). Other recommendations included providing transportation assistance (8.8%), improving overall meal quality, menu options and timings (7.9%), making the congregate meal services more accessible by removing physical access barriers (5.2%), and improving congregate meal programming (4.9%) activities to engage the seniors creatively.

The survey was launched in August 2020 when the COVID-19 pandemic was at its peak. Sites either had to close down or operate at limited capacity. In acknowledgment of the COVID-19 context, 38.2% of respondents suggested that social distancing, wearing masks, and good hygiene practices should be incorporated to make the CMPs a success. An additional 25.3% of respondents suggested contactless home delivery of meals, and 11.7% suggested thoughtful outreach initiatives centered on virtual contact. Other suggestions included limiting participation to small groups (4.9%), allowing drive-thru meal pick-up (3.6%), and screening/testing of staff and participants (1.9%).

## Discussion

Community healthcare providers serve as facilitators of knowledge about, and access to, CMPs for seniors across the state of Texas. Consequently, an assessment of the providers’ referral processes and perceptions was critical in understanding how to increase senior access to and participation in CMPs. The results presented here contribute to valuable understanding of the CMP referral process as well as the motivators for and facilitators of participation in Texas CMPs by older Americans.

The majority of providers who responded to the survey indicated that their perceptions of CMPs were positive. Most providers rated the CMPs from good to excellent and indicated that CMPs were beneficial for decreasing the food insecurity and social isolation of seniors and fulfilling their nutritional and dietary needs by providing them access to meals in social settings. The majority also rated the quality of CMPs as good, very good, or excellent. The perceptions of physicians and nurses as indicated by survey results align with the general purpose of LTSS and literature describing the nutritional value offered by the CMPs. Specifically, LTSS aims to provide care and support to older adults with activities of daily living (ADLs) in their natural settings (homes or community-based settings) to help them live more independently and freely (Centers for Medicare & Medicaid Services n.d.; Reaves and Musumeci 2015). Studies have shown that CMPs help alleviate food and nutrition insecurity by providing consistent access to a healthy diet, leading to good health (Beasley et al. 2018; Hartline-Grafton 2019; Walton et al. 2020). Studies also show that visiting these sites daily allows older Americans to interact with peers who may share similar interests and behaviors with them, resulting in better mental health, lower depression and loneliness, and greater socialization (Lloyd and Wellman 2015; Mabli et al. 2017; Greenlaw 2018).

Research has shown that congregate meal participants have lower food insecurity rates, more favorable socialization outcomes, and higher diet quality compared to non-participants (Mabli et al. 2017). Existing studies suggest that the congregate nature of these meal programs is key to specific socialization outcomes, as home-delivered meal participants were found to have similar or less favorable socialization outcomes than CMP participants. In comparison to non-participants, both CMP participants and home-delivered meal participants had a higher diet quality. However, both home-delivered meal participants and non-participants had similar rates of food security.

Providers who refer patients to CMPs cited many of these benefits as reasons for referral, specifically indicating that they predominantly refer patients *to enable patients to have access to nutritional meals*, *to enable patients the opportunity to socialize with others and address their social integration needs*, and *to enable patients the opportunity to participate in activities at locations where meals are served.* However, although many providers recognize the utility of CMPs for the overall health of the older American population, many indicated that they had never referred patients to congregate meal programs. The data further indicated that some providers have connected patients to a service coordinator who then determined the type of services that the patient needed. This connection to the coordinator may or may not have resulted in a CMP referral.

As evidenced by the survey responses, there is a clear lack of awareness of the services provided by CMPs which contributes to the underutilization of CMPs as a referral option to enhance the general nutritional health and well-being of older adults. Furthermore, of the providers surveyed, physicians were less aware of CMP services compared to nurses. A review of the referral processes for the various providers surveyed suggests that nurses may be more likely to be involved in the referral process than physicians, as some referral processes described require nurses to serve as referral coordinators.

Research suggests that physicians’ lack of knowledge of community services and programs is not a phenomenon unique to CMPs. A study of physician awareness of enhanced prenatal services (EPS) in Michigan found that although upwards of 90% of physicians indicated that EPS would be beneficial to their patients, 84% were not amply familiar with EPS, and 60% had not personally referred patients to EPS (Raffo et al. 2014). Fifty-four percent were also unaware of whether someone else in the office handled referrals to these programs (Raffo et al. 2014).

Studies show that food-insecure individuals are less likely to take advantage of community-based food programs because of limited knowledge regarding eligibility and fears around government program enrollment (Marpadga et al. 2019). Healthcare providers are in a position to coordinate, promote, and educate patients about nutrition and food access, as well as advocate and provide social support to enable patients to attain food security by referring them to CMPs. Although studies have shown concerns with implementing a standardized referral process due to limited resources and time spent with patients (Keller et al. 2007; Pooler et al. 2018), addressing these barriers by making food insecurity a primary health concern and programs such as CMPs an integral part of the referral process will help improve the overall health and well-being of Texas’s older adult population by increasing both provider and client awareness of the CMP programs.

### Reasons for not participating

Although healthcare providers may help facilitate access to congregate meal programs, participation is ultimately at the discretion of the clients. Across the state of Texas, participation in CMPs is not commensurate with the number of senior citizens who could benefit from such programs. Healthcare providers most frequently suggested that issues pertaining to transportation and the accessibility of congregate meal sites may contribute to the lack of utilization of CMPs. In an evaluation of a program similarly targeting older adults experiencing social isolation (Senior Centers Without Walls), seniors most often cited the ease in accessing the program from their home as a reason for participating. The authors go on to suggest that removing barriers related to physically accessing services, such as transportation, may facilitate greater participation in programs targeting seniors (Levasseur et al. 2015; Newall and Menec 2015). To this end, healthcare providers included in the present study suggested that religious organizations such as churches, senior housing, and local community halls may be the most accessible locations for the older adult population.

Beyond the physical accessibility of CMP sites, healthcare providers also frequently suggested that the stigma associated with free meals may contribute to the decline in CMP participation. As with many aspects of health and aging, there are various age-related stigmas and financial hardships which may prevent individuals from seeking support. Studies show that stigmas pertaining to mental health, financial assistance, and physical ability negatively influence participation in and acceptance of support services (Dobbs et al. 2008; Roth et al. 2016). Promoting CMPs through community outreach and partnerships, strategic marketing, and educational programming has the potential to eliminate these stigmas associated with participation (Hoerr et al. 2016).

Although to a lesser extent, some healthcare providers indicated that older adults may be deterred from participating in CMPs by a lack of food variety, lower quality of food being offered, and dietary restrictions that limited the types of foods they could eat. With the growing multicultural landscape and changing palate of older Americans, congregate meal providers need to cater to the different dietary practices and choices preferred by older Americans in order to make the CMPs a success. Studies have shown that most Asians are lactose-intolerant and are unable to consume meals with dairy components (Namkee 2002; Porter and Cahill 2015), highlighting the need for a dairy-free meal option for lactose-intolerant clients. Texas CMPs need to carefully integrate ethnic recipes that help facilitate wellness through diverse cultural food traditions. The dietary habits and nutritional needs of Hispanic seniors need special attention because they comprise more than 39% of the Texas population according to the 2020 census (Cuy Castellanos 2015). Studies show that Hispanics (Ura et al. 2021) face nutrition-related chronic diseases such as diabetes and obesity (Gray et al. 2005), highlighting the need for healthy meal options that are low in saturated fat, salt, and starchy vegetables and high in nutrients (Lin et al. 2003).

### Limitations

The present study produced important insights into the healthcare provider referral process for CMPs in Texas. However, there are limitations to the interpretation of these data based on the sources of the data and characteristics of the sample. The sample was relatively small, consisting of 412 healthcare providers who primarily serve urban communities. Consequently, the perspectives of the providers, as they pertain to patient challenges and motivations, are likely embedded within the context of urban community environments. Some of their concerns may not be directly applicable to rural environments.

The data presented may also be limited by potential differences in the participants’ interpretation of some survey items. Specifically, many healthcare providers claimed that they had never referred patients, but there may have been some confusion about what constituted a “patient referral.” For example, some physicians mentioned that they had never referred patients to CMPs but also mentioned that patients are referred to a social worker who handles care coordination and referrals. In other words, some participants did not consider referring the patient to a third party who handles the referral to be an example of themselves referring patients to CMPs, whereas others did.

Beyond specific aspects of the patient referral process for CMPs, healthcare providers were also asked to express their opinions of why older adults do not participate in congregate meal programs. As a secondary source of information, the speculations of healthcare providers may not accurately reflect the perceptions of their patients. As studies have shown, there are often gaps in alignment between the perceptions of healthcare providers and their patients (Coppola et al. 2014; Goldberg and Shorten 2014; Abuosi 2015; Williams et al. 2016). Consequently, although the opinions of healthcare providers are valuable sources of information to use in developing and modifying targeted initiatives and programs, considering their perceptions alongside patient perceptions would better speak to the motivations and challenges of patients. Although the TCMI older American survey (*Texas Congregate Meal Initiative* n.d.) revealed low-quality meals, dietary restrictions, and stigma attached to attending CMPs as top barriers responsible for a decline in meal program attendance, more work that compares the perceptions of patients with those of healthcare providers is needed.

### Practical implications

Survey results revealed that only 39.8% (164) of the respondents had referred their patients to CMPs, and that many healthcare providers had never referred patients to CMPs because they were unaware of the programs. Due to the number of physicians who were unaware of the existence of CMPs in the areas they served, extensive marketing efforts need to be made to educate the healthcare provider community about the CMPs offered in their areas. In turn, increased provider awareness may lead to an increase in CMP referrals and participation rates by Texas seniors. To increase advocacy for these programs, program administrators should also consider outreach involving word of mouth, places of worship, and media platforms such as radio and television, as these places were frequently reported as key vehicles of information for older adults by the healthcare providers surveyed.

Additionally, the quality and variety of meals offered may need to be improved to attract more seniors to CMPs. Opinions about CMPs revealed that 15.3% of the respondents indicated that the quality of the programs varies: some are good, and some are not. Furthermore, 26.0% of the respondents stated that older adults have dietary restrictions that may limit their participation. Consequently, a greater variety and consistent quality of meals may lead to increased CMP participation.

### Future directions

The present survey study spotlighted the urgent need for healthcare provider education about the LTSS offered in their communities. The study showed that many of the healthcare providers surveyed were unaware of the congregate meal programs offered in their communities. This indicates that additional research is needed so that decision-makers understand how to best disseminate information to healthcare providers to increase the referrals and improve the referral mechanisms and use of nutritious meal-oriented public service programs like CMPs by the aging populations who can most benefit from them.

Furthermore, as previously mentioned, interpretation of data presented through this survey study is limited by the secondary nature of the data. A survey of healthcare provider perceptions about the motivations and experiences of older adults is beneficial for generating ideas for how to address client participation and increase referrals by healthcare providers; however, a direct assessment of client motivations to participate in CMPs and challenges associated with participation needs to be considered alongside provider perceptions in order to best address these challenges and increase senior motivation to participate. Future research focusing on LTSS referral mechanisms for seniors should focus on understanding the varying perspectives among various older adult age groups to better serve the older adult population. Data collected by the Public Policy Research Institute (PPRI), Texas A&M University, as part of a larger congregate meal initiative seeks to address this issue by surveying older Texans’ perceptions about CMPs, but additional research is needed to understand the systemic connections between provider referral mechanisms and older adults’ preferences, motivations, and needs for food security and nutrition.

The older Texan population, similar to the older adult population across the United States, is rapidly changing. Members of the new aging population are categorized as Baby Boomers (people born from 1946 to 1964), and research suggests that Baby Boomers' primary motivator for maintaining a healthy lifestyle is to remain independent (Kahana and Kahana 2014). Providing educational programming integrating wellness and lifestyle-based interventions and activities may help address the needs specific to this generation (Hoerr et al. 2016). Among the independently living older Americans, several other subgroups are studied today, and the needs of seniors 60 to 70 years of age differ distinctly from those of seniors belonging to the 71–80-year or 81–90-year age range. Many seniors are working longer, caring for younger grandchildren, interested in part-time positions and flexible hours, and continuing to work for various social, physical, and psychological benefits. It is important that future research continues to study the older adult population as it ages, as needs of future generations are likely to differ from the needs of previous generations of older adults. The evolving data produced by continuous research may aid in attracting older adults to more targeted programs and services that align with their interests.

In addition to the changing interests of the aging population, demographic changes are also taking place. There are more people migrating to the United States each year (Mower 2008; Porter and Cahill 2015), which is causing the older adult population to become increasingly diverse. Per ACL’s 2020 Profile of Older Americans, “racial and ethnic minority populations have increased from 9.6 million in 2005 (19% of the older adult population) to 19.6 million in 2020 (25% of older adults) (NANASP and the National Resource Center on Nutrition and Aging 2019). Many members of religious communities such as Jewish and Muslim communities have dietary restrictions and may only eat kosher- and/or halal-certified meat. There is a growing need to cater to an ethnically diverse older American population as the ethnic and religious demography of the older American population becomes increasingly diverse (Mower 2008; Porter and Cahill 2015). Consequently, it is imperative that future research aid healthcare providers in understanding the health and economic benefits of serving the diverse nutritional and dietary needs of the older American population. Likewise, there is a need to employ staff at the meal sites who represent the populations they are serving. Employing staff who are linguistically and ethnically representative of the population they serve has the potential to result in higher CMP participation (Porter and Cahill 2015).

## Conclusion

LTSS services accessed through referrals by Texas healthcare providers have a great impact on the health, nutrition, and social well-being of aging Texans. The providers’ awareness of services and a systematic referral process to connect aging adults to CMPs is integral to directly improving and sustaining their health. Provider awareness about the CMPs and a seamless referral process integrating the CMPs offered in their community are valuable components for support services that will be beneficial to the seniors the providers serve. The healthcare provider's awareness and perception of CMPs has a critical impact on the aging population’s access to obtain food security and greater quality of life due to improved health.

CMPs have resulted in positive outcomes such as nutritious meals, increased nutritional education, and improved mental health and well-being for seniors due to social interaction opportunities offered through CMP activities and programing in addition to the access to meals. Healthcare providers in Texas recognize the value of connecting seniors to CMPs and have a good idea of what might work to motivate the seniors to participate in CMPs. However, most health providers are unaware of the CMPs in their communities, and many do not have CMPs as an option in their LTSS referral process. The survey provides pivotal information for Texas decision-makers and aging stakeholders that may help strategize awareness-building and spotlight the value of a well-coordinated referral process so that healthcare providers in Texas can have CMPS in their LTSS referral list. Serious attention to such awareness-building and a reworked referral process will ultimately help address the problem of declining participation in Texas CMPs amidst an increasing older Texan population with food security needs.

## Data Availability

Data that support the findings of the project are not publicly available but can be shared by submission team with the journal if necessary.

## Code availability

Submission team can share code books if necessary with journal.
